# Alkylation or Silylation for Analysis of Amino and Non-Amino Organic Acids by GC-MS?

**DOI:** 10.3390/metabo1010003

**Published:** 2011-01-17

**Authors:** Silas G. Villas-Bôas, Kathleen F. Smart, Subathira Sivakumaran, Geoffrey A. Lane

**Affiliations:** 1 Centre for Microbial Innovation, School of Biological Sciences, The University of Auckland, Private Bag 92019, Auckland, New Zealand; 2 AgResearch Limited, Grasslands Research Centre, Tennent Drive, Palmerston North 4442, New Zealand

**Keywords:** Microbial metabolomics, metabolite profiling, metabolome, derivatization, gas chromatography, mass spectrometry, chroroformates, TMS

## Abstract

Gas chromatography–mass spectrometry (GC-MS) is a widely used analytical technique in metabolomics. GC provides the highest resolution of any standard chromatographic separation method, and with modern instrumentation, retention times are very consistent between analyses. Electron impact ionization and fragmentation is generally reproducible between instruments and extensive libraries of spectra are available that enhance the identification of analytes. The major limitation is the restriction to volatile analytes, and hence the requirement to convert many metabolites to volatile derivatives through chemical derivatization. Here we compared the analytical performance of two derivatization techniques, silylation (TMS) and alkylation (MCF), used for the analysis of amino and non-amino organic acids as well as nucleotides in microbial-derived samples. The widely used TMS derivatization method showed poorer reproducibility and instability during chromatographic runs while the MCF derivatives presented better analytical performance. Therefore, alkylation (MCF) derivatization seems to be preferable for the analysis of polyfunctional amines, nucleotides and organic acids in microbial metabolomics studies.

## Introduction

For analysis of volatile compounds, gas-chromatography (GC) coupled to mass spectrometry (MS) allows high analysis throughput at relatively low cost. GC-MS is the most popular analytical technique in metabolomics today because it separates complex metabolite mixtures with high efficiency. Compound identification by GC-MS is also easier due to the high reproducibility of fragmentation patterns in electron impact (EI) ionization mass spectra, and the ready availability of libraries of spectra [1,2]. However, most naturally occurring metabolites are not sufficiently volatile to be analyzed directly on a GC system. Chemical derivatization of the metabolites is therefore required, and high analysis throughput by GC-MS relies on fast and efficient derivatization techniques [1,2].

**Figure 1.**
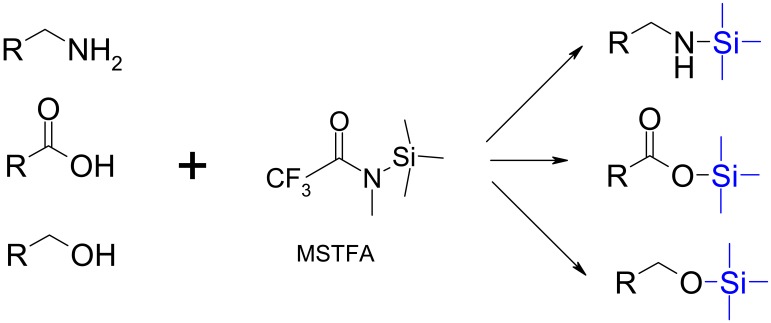
Overall scheme of chemical derivatization of metabolites by silylation using trimethylsilyl derivatives, e.g. N-methyl-N-(trimethylsilyl)trifluoroacetamide (MSTFA). The formation of derivatives from MSTFA involves the displacement of an N-methyltrifluoroacetamide leaving group by the analyte.

A large number of derivatization methods for analysis of metabolites have been reported, but only a few are currently used in metabolomics [1,2]. Silylation of organic compounds is the classical and most widely used derivatization procedure for metabolome analysis by GC-MS (Figure 1) [1-6]. Sugars and their derivatives (sugar alcohols, amino sugars, and others) are the class of metabolites most efficiently derivatized by silylation [1,2,6]. However, some important primary cell metabolites such as the amino acids and some organic acids produce relatively unstable silylated derivatives [7-9], which call for alternative derivatization methods for an efficient analysis of these compounds. Furthermore, silylation reactions require anhydrous reaction conditions, and therefore, the samples have to be completely free of water, and the reagent mixture in silylation reactions is not separated from the derivatives, thus, the sample injected into the chromatographic column contains derivatives and residual reagents as well as non-derivatized involatile compounds. This is especially problematic for complex biological samples such as extracellular culture media and body fluids, which contain large amounts of non-derivatizable compounds that may damage a capillary GC column.

**Figure 2.**
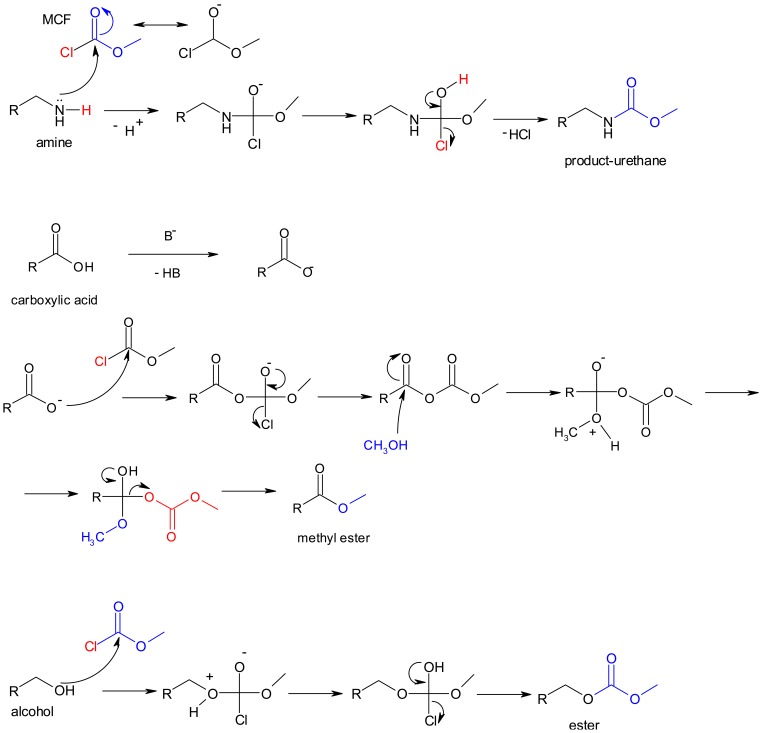
Overall scheme of chemical derivatization of metabolites by alkylation using methyl chloroformate (MCF). Mainly amino and non-amino organic acids are derivatized by this technique, but some amines and alcohols can also be derivatized as shown below.

Alkylation is an alternative derivatization reaction that can be used in metabolite analysis by GC and GC-MS [1,2,10-12]. This method is primarily used for derivatization of polyfunctional amines and organic acids, and a novel alkylation protocol based on methyl chloroformate (MCF) derivatives has been reported that enables the GC-MS analysis of over a hundred amino and non-amino organic acids simultaneously (Figure 2) [13-15]. Of the 600 metabolites documented by Förster *et al.* [16] in a genome-wide metabolic model for yeast, approximately 40% are amines, amino acids or organic acids (not including fatty acids), which play crucial roles in central carbon metabolism and amino acid biosynthesis. Unlike silylation, the alkylation derivatization offers instantaneous reaction without heating or water exclusion, lower reagent costs, and easy separation of the derivatives from the reaction mixture, which causes less damage to the GC-capillary column. Therefore, MCF derivatization is the best candidate to be used in parallel with silylation in order to achieve the goal of metabolomics that is the detection and analysis of as many metabolites as possible in biological samples.

In this work, we report a comparative study on the analytical performance of the most popular silylation reaction (methoxymation followed by per-trimethylsilylation – referred hereafter as TMS) and the previously reported alkylation protocol based on MCF derivatization for the simultaneous analysis of amino and non-amino organic acids as well as nucleotides. We compare here the stability of the derivatives, the reproducibility of derivatization, the dynamic range of detection, linearity, matrix effect of both derivatization methods, as well as the performance of both methods during analysis of microbial-derived metabolites in culture media using a standard GC-MS platform.

## Experimental

### Chemicals

Methanol, sodium hydroxide, chloroform and sodium sulfate used for chemical derivatization were all of analytical grade and purchased from different suppliers. The derivatization reagents methyl chloroformate (MCF), N-methyl-N-(trimethylsilyl)trifluoroacetamide (MSTFA), methoxylamine hydrochloride and pyridine as well as the isotope-labeled internal standard L-alanine-2,3,3,3-d_4_ and all metabolite standards were obtained from Sigma-Aldrich (St. Louis, MO, USA).

### Standards

We selected representative compounds from different classes of metabolites for this study (Table 1). Stock solutions of standards (except fatty acids) were prepared in water at a final concentration of 10 mM. Stock solutions of the fatty acids myristate and palmitate were prepared in methanol at a final concentration of 10 mM. The standard mixtures were dried under vacuum using a SAVANT speed vacuum (Speed Vac® Plus) SC110A (Savant Instruments, Inc., Holbrook, NY, USA) prior to chemical derivatization.

**Table 1.** List of metabolite standards used to assess the analytical performance of chemical derivatization.

**Metabolite class**	**Metabolite representatives and abbreviations**
Neutral amino acids	Alanine (ALA), valine (VAL), glutamine (GLN)
Basic amino acids	Lysine (LYS)
Acidic amino acids	Glutamate (GLU), aspartate (ASP)
Aromatic amino acids	Phenylalanine (PHE), tryptophan (TRP)
Sulfur-containing amino acids	Methionine (MET), cysteine (CYS)
Monocarboxylic acids	2-Hydroxybutyrate (2HB), lactate (LAC), phosphoenolpyruvate (PEP)
Dicarboxylic acids	Succinate (SUC), fumarate (FUM), oxaloacetate (OAA)
Tricarboxylic acids	Citrate (CIT), isocitrate (ICI)
Aromatic carboxylic acids	*p*-Coumarate (COU), ferulate (FER)
2-oxo-acids	2-oxoglutarate (AKG), 3-methyl-2oxovalerate (OIVAL)
Fatty acids	Myristate (MYR), palmitate (PAL)
Nucleotides	NAD^+^, NADP^+^
Internal standard	L-Alanine-2,3,3,3-d_4_ (D4-ALA)

### Spent microbial culture

Spent culture of *Clostridium proteoclasticum* strain B316^T^ [17] grown anaerobically for 24 hours on a modified rumen bacteria medium 330 published by DSMZ (Brounschweig, Germany, www.dsmz.de/media/med330.htm) was used to assess matrix effects on the derivatization reactions. The medium (before microbial inoculation) contained peptone (2 g/L), yeast extract (2 g/L), haemin (1 mg/L), resazurin (1 mg/L), cysteine-HCl (0.25 mg/L), K_2_HPO_4_ (0.6 g/L), Na_2_CO_3_ (4 g/L), supplemented with a mixture of volatile fatty acids (10 mL/L), mineral solution (75 mL/L), and glucose (8 g/L), Birchwood xylan (Sigma) (2 g/L), and apple pectin (Sigma) (2 g/L) as the major substrates.

Spent culture of five different strains of *Acidovorax temperans* grown aerobically for 12 hours on R2A medium (EMD Chemicals, Inc. Darmstadt, Germany), which contained peptone (1.5 g/L), glucose (0.5 g/L), starch soluble (0.5 g/L), sodium pyruvate (0.3 g/L), buffers (0.3 g/L), MgSO_4_ (0.024 g/L); was used to assess the performance of each derivatization on real biological samples. 1 mL-samples of spent bacterial medium were prepared by filtering exponentially growing *A. temperans* cultures through a 0.22 μm membrane filter (n = 9). Appropriate internal standard (2,3,3,3-d4 alanine) was added to each sample and the samples were then freeze-dried prior to derivatization.

### Sample derivatizations

The TMS derivatization (when not indicated otherwise) was based on the optimized protocol described by Villas-Bôas *et al.* [6]. In summary, the dried samples were resuspended in 80 μL of methoxyamine hydrochloride solution in pyridine (2 g/100 mL) and incubated in a domestic microwave oven for 2.8 min, with multimode irradiation set to 400 W and 30% of exit power. MSTFA (80 μL) was then added to each sample followed by 3.0 min incubation in a microwave oven as described above.

The MCF derivatization was performed according to Villas-Bôas *et al.* [13]. In summary, the dried samples were resuspended in 200 μL of sodium hydroxide solution (1 M) and mixed with 34 μL of pyridine and 167 μL of methanol. 20 μL of MCF was added to the reagent mixture followed by vigorous mixing for 30 s using a vortex. Another 20 μL of MCF was added to the reactive mixture followed again by vigorous mixing for another 30 s. To separate the MCF derivatives from the reactive mixture, 400 μL of chloroform was added to the mixture and then mixed vigorously for 10 s followed by the addition of 400 μL of sodium bicarbonate solution (50 mM) and vigorous mixing for an additional 10 s. The upper aqueous layer was discarded and the chloroform phase was subjected to GC-MS analysis.

### GC-MS analysis and compound identifications

GC-MS analysis was performed with a Shimadzu GCMS-QP2010 system, equipped with a quadrupole mass selective detector on electron impact (EI) mode operated at 70 eV. The column used for all analyses was a ZB1701 (Zebron, Phenomenex), 30 m × 250 μm i.d. × 0.15 μm film thickness. The MS was operated in scan mode (start after 4.5 min, mass range 40-650 a.m.u. at 0.15 s/scan). The parameters for separation and analysis of TMS and MCF derivatives are described in Villas-Bôas *et al.* [6] and Smart *et al.* [15], respectively. For compounds forming more than one major derivative, the most intense peak was selected for quantitation. We have used the Automated Mass Spectral Deconvolution and Identification System (AMDIS) to identify compounds present in each sample based on mass spectra and retention times against our in-house MS library of spectra. AMDIS is a software freely distributed by the National Institute of Standards and Technology and has been largely applied to metabolomics.

### Repeatability of the GC-MS equipment

To assess the repeatability of the analytical instrument (GC-MS), we derivatized two different concentrations of standards known to produce stable derivatives by both derivatization methods and we analyzed the same sample 6 times in sequence. The repeatability was assessed by determining the relative standard deviation (RSD) of the GC-peak area, using Equation (**1**), of each metabolite derivative between the 6 analyses.

(1) RSD=SD/mean×100

### Stability

The standard mixture containing all metabolites listed in Table 1 was derivatized in two different concentrations (n = 2) and immediately injected into the GC-MS. The same samples were re-injected after 24, 48 and 72 hours. The stability of the metabolite derivatives was assessed by determining the relative standard deviation (RSD) of the GC-peak area, Equation (**1**), of each derivative within 72 hours.

### Repeatability of derivatization

To assess the repeatability of the derivatization reactions we derivatized 6 replicates samples of the standard mixture listed in Table 1 in two different concentrations. Each sample was injected into the GC-MS immediately after derivatization. The repeatability was assessed by determining the relative standard deviation (RSD) of the GC-peak area, using Equation (**1**), of each metabolite derivative between the 6 replicate samples.

### Matrix effect

To evaluate the effect of the sample matrix on the derivatization reaction we spiked the standard mixture listed in Table 1 in spent microbial culture medium samples (n = 6) and we compared the response factor and recoveries of the different metabolite derivatives compared to the mixture of standards alone. Each sample was injected into the GC-MS immediately after derivatization. The recovery was calculated by comparing the GC-peak area of each metabolite derivative when analyzed in a standard mixture alone or spiked on spent microbial culture medium. The contribution by metabolites genuinely present in the spent culture medium was subtracted from the final results.

### Derivatization of biological samples

To evaluate the performance of each derivatization technique on real biological samples we derivatized spent culture medium samples (n = 9) of five different strains of *Acidovorax temperans* using both derivatization techniques. The methods were compared based on the number of metabolites detected and identified as well as on their ability to discriminate the different *A. temperans* strains. GeneSpring MS 1.2 software (Agilent Technologies, Santa Clara CA, USA) was used for data mining and multivariate data analysis.

## Results

### Repeatability of GC-MS analysis

As a baseline for comparing the two derivatization techniques, we first determined the repeatability of our measurements with our GC-MS equipment, including factors such as variation in injection volumes, performance, *etc*. Samples containing a mixture of compounds that produce stable derivatives by both silylation and alkylation were derivatized and injected six times into the GC-MS. Table 2 presents the variability observed between the six analyses. Excellent performance of the instrument was clearly demonstrated for both silylated and alkylated derivatives with relative variability below 10% (except for cysteine 50 *p*mol, MCF, 11.5%).

### Stability of different derivatives

**Figure 3.**
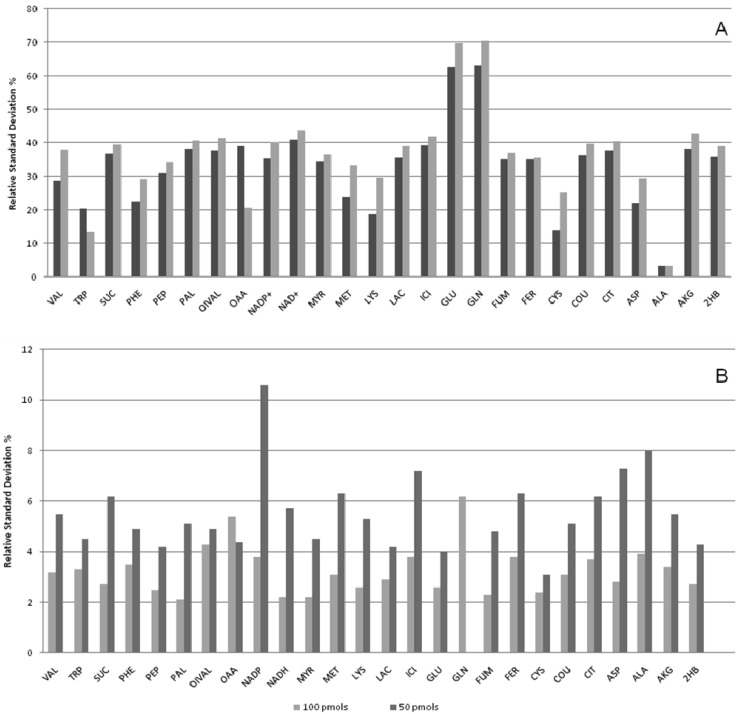
Relative standard deviations of the GC peak areas of metabolite derivatives analyzed every four hours during 72 hours. (**A**) Silylation (TMS); (**B**) Alkylation (MCF). Legend shows concentration of metabolites per samples. See Table 1 for metabolite abbreviations.

**Figure 4.**
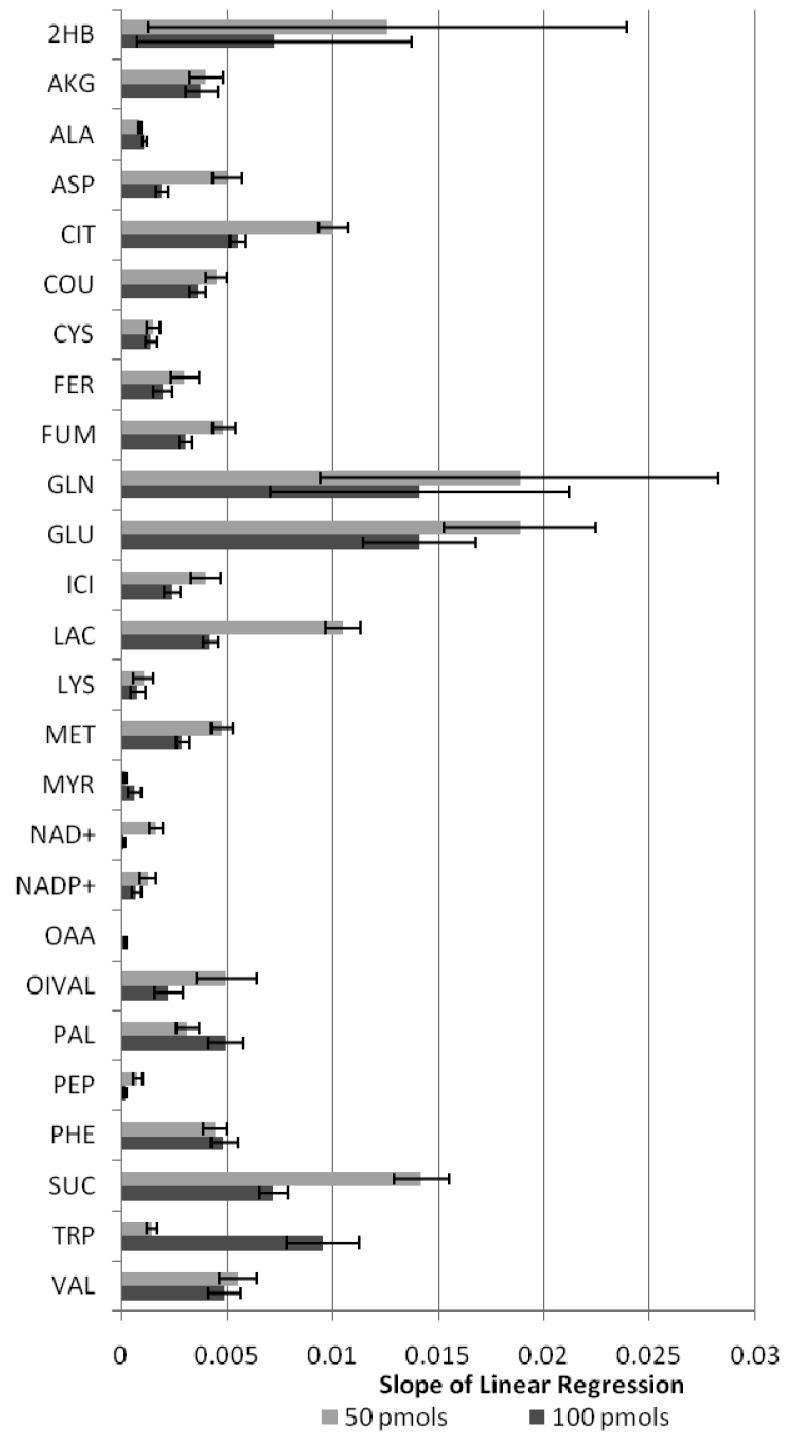
Slope values obtained from the linear regression of the GC peak areas of silylated (TMS) metabolite derivatives analyzed four times during 72 hours. Legend shows concentration of metabolites per samples. See Table 1 for metabolite abbreviations.

The stability of metabolite derivatives is an important parameter for derivatized samples that may have to wait hours in a queue before injection. Figure 3 presents the variability of metabolite level data within 72 hours for both derivatization techniques tested. Except the amino acid alanine, all silylated derivatives presented a pronounced variability within 72 hours compared to alkylated compounds (Figure 3A). For all compounds the yield of the derivative increased (Figure 4) indicating the silylation reaction was not driven to completion. With only one exception in the lower concentration mixture, all MCF derivatives were found to be remarkably stable over 72 hours (3 days) at room temperature (RSD < 10%) (Figure 3B). The internal standard in the samples was an isotope-labeled alanine, and evidently this could correct for the variation of silylated alanine levels. However, other silylated derivatives showed variable degrees of instability. Therefore, unless we are able to include an isotope-labeled standard for each metabolite analyzed in the samples, silylated-derivatives of amino and non-amino organic acids should be analyzed immediately after the derivatization reaction, or at least the interval between derivatization and analysis should be held constant. The MCF derivatized samples, on the other hand, were stable, and thus are not required to be injected directly after derivatization, which makes this method more robust for batch analysis of amino and non-amino organic acids.

**Table 2.** Repeatability (RSD) of the GC-MS instrument for some stable metabolite derivatives.

**Metabolite**	**RSD % (n = 6)**			

	**Silylation (TMS)**		**Alkylation (MCF)**	

	**Concentration (pmol/sample)**			

	**100**	**50**	**100**	**50**
ALA	0.4	0.7	1.9	8.0
ASP	2.0	3.1	0.9	6.7
COU	6.1	4.6	2.1	6.5
CYS	2.0	4.4	6.2	11.5
FUM	3.6	1.7	1.1	5.4
LAC	3.9	2.9	0.7	6.1
OIVAL	5.2	3.0	0.4	5.6
PHE	3.8	4.5	1.4	6.7
SUC	4.6	3.1	1.3	5.5
VAL	1.6	1.8	2.1	5.4
2HB	3.8	2.5	2.4	6.4

See Table 1 for metabolite abbreviations.

### Repeatability of derivatization reaction

**Figure 5.**
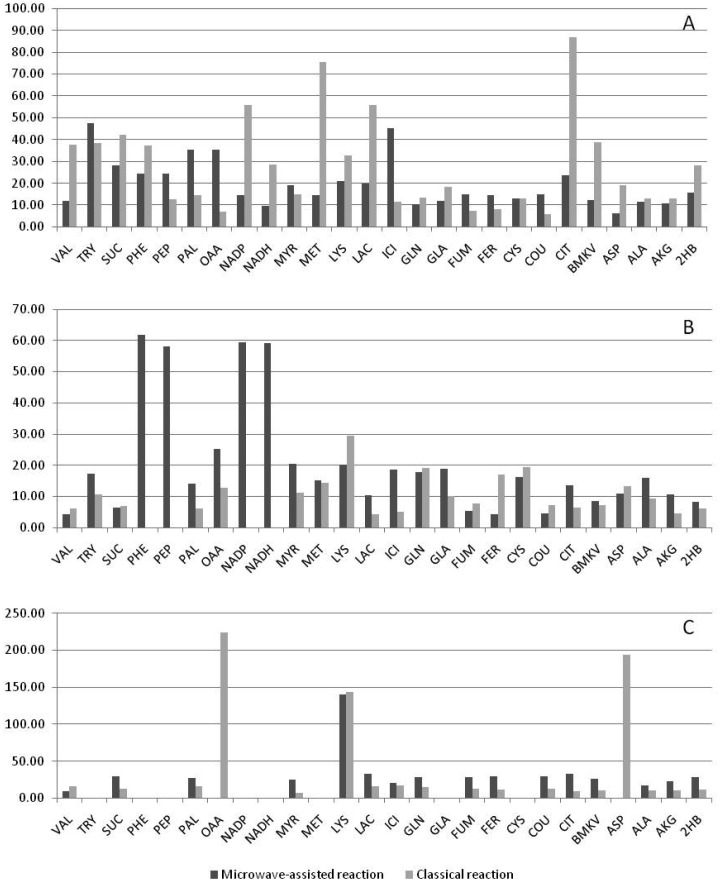
Reproducibility (RSD) of the TMS derivatization for several metabolites using two different reaction protocols: Microwave-assisted reaction according to Villas-Bôas *et al.* [6], and the classical reaction according to *Roessner et al.* [4]. (**A**) 100 *p*mols/sample; (**B**) 50 *p*mols/sample; (**C**) 25 *p*mols/sample. See Table 1 for metabolite abbreviations.

The median variability of the raw peak areas of MCF derivatives at both concentrations of 26 standards tested was 8.2% and the maximum variability 11.60% or less, except for the amino acid glutamine (RSD ∼ 20%) (Table 3). The TMS derivatives, on the other hand, showed considerably higher variability particularly for amino acids and nucleotides (Table 3) (overall median 32.8%, maximum > 25% for 13 of 26 compounds). Oxaloacetate and tryptophan were not detected in any mixture derivatized by TMS (Table 3). To check whether the high variability of TMS derivatization could be attributed to our modified protocol, which makes use of microwave heating to increase the reaction throughput [6], we compared the reproducibility of TMS derivatization using both this and the classical protocol [4]. Figure 5 presents the variability of both TMS derivatization protocols. The variability of TMS derivatization was slightly lower for the classical than for the microwave derivatization protocol (median RSD 12.9% classical, 18.2% microwave, maximum > 25% for 13 compounds classical, 18 compounds microwave), but not comparable with MCF, which in this study was a more reproducible derivatization technique for analysis of amino and non-amino organic acids than TMS derivatization.

**Table 3.** Reproducibility (RSD) of the derivatization efficiency for several metabolites.

**Metabolite**	**RSD % (n = 6)**			

	**Silylation (TMS)**		**Alkylation (MCF)**	

	**Concentration (pmol/sample)**			

	**100**	**50**	**100**	**50**
ALA	5.7	12.1	7.7	5.2
ASP	5.9	17.7	9.4	5.7
AKG	10.4	9.9	10.8	5.6
CIT	10.8	9.3	8.7	4.9
COU	9.1	44.2	9.1	6.8
CYS	10.8	9.3	11.4	10.1
FER	9.2	11.9	8.4	8.2
FUM	10.3	11.9	7.9	9.1
GLN	44.9	34.4	21.8	19.0
GLU	20.0	14.8	4.7	4.7
ICI	35.1	24.0	10.0	14.7
LAC	20.6	8.8	8.3	6.0
LYS	25.0	25.7	6.7	5.4
MET	37.6	75.4	3.9	6.4
MYR	12.4	17.8	6.8	6.0
NAD+	155.0	nd	11.0	7.1
NADP+	38.8	244.9	10.6	11.6
OAA	nd	nd	9.1	8.5
OIVAL	31.4	19.7	7.2	4.8
PAL	8.6	22.4	8.2	6.7
PEP	41.5	244.9	10.9	8.8
PHE	34.4	22.5	6.1	8.0
SUC	10.8	14.2	5.2	7.0
TRP	nd	nd	6.6	9.6
VAL	18.0	16.8	7.7	5.2
2HB	19.7	4.5	8.7	7.2

See Table 1 for metabolite abbreviations. nd, not detected

### Dynamic and linearity ranges

The dynamic range for detection of MCF derivatives by GC-MS (8–100 fold) was found to be somewhat wider than for TMS derivatives (5–63 fold). Due to their instability we could not determine the dynamic range for several TMS-derivatized metabolites such as NAD^+^, NADP^+^, phosphoenolpyruvate, and tryptophan (Table 4). Similarly, the linear range for all MCF derivatives (except palmitate) was relatively wider compared to TMS derivatives (Table 4). Therefore, we conclude that the MCF derivatization method is more appropriate for a quantitative analysis of amino and non-amino organic acids.

### Matrix effect

**Figure 6.**
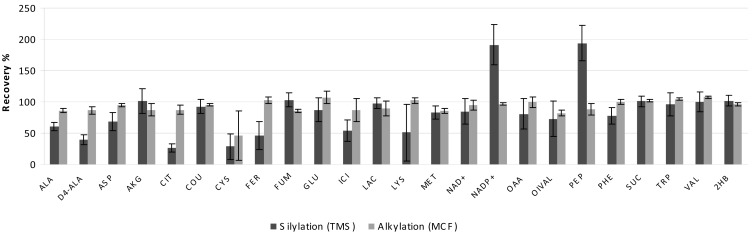
Recovery of the metabolite standard spiked on a spent microbial culture medium after silylation and alkylation derivatizations. See Table 1 for metabolite abbreviations.

The susceptibility of the analytical performance of the two derivatization techniques to interference posed by the sample matrix components was assessed by derivatizing standard mixtures spiked into a complex biological sample (spent microbial culture medium). By comparing the response factors of each metabolite derivative in a mixture of pure standards with the response factors of the same standards spiked into spent microbial culture media (Figure 6), we observed that both derivatization techniques are affected by the matrix of the sample. The response factor decreased for several TMS and MCF derivatives; however some TMS derivatives appeared to be more susceptible to matrix effect than MCF derivatives (e.g.; alanine, aspartate, citrate, cysteine, ferulic acid, isocitrate, and lysine). Interestingly, the phosphorylated metabolites NADP and phosphoenolpyruvate presented a higher response factor when spiked in a spent culture medium (Figure 6). These compounds were not detected in the spent microbial culture medium alone and, therefore, the silylation of these metabolites must be somehow favored in a complex sample matrix.

**Table 4.** Linearity and dynamic range of several metabolite standards.

**Metabolite**	**Silylation (TMS)**			**Alkylation (MCF)**		

	**Dynamic range pmol/sample**	**Linearity pmol/sample**	r^2^*	**Dynamic range pmol/sample**	**Linearity pmol/sample**	r^2^*
ALA	≤ 10–400	10–80	0.97	25–200	25–200	1.00
ASP	40– ≥ 630	40–80	0.81	≤ 10 ≥ 400	25–100	0.98
AKG	40 ≥ 630	NL	-	25 ≥ 2000	25–200	0.98
CIT	≤ 10–400	10–80	0.98	≤ 10– ≥ 400	25–200	0.97
COU	25–500	20–80	0.98	≤ 10– ≥ 400	25–150	0.98
CYS	40– ≥ 400	40–80	0.77	25– ≥ 1000	25–200	0.98
FER	40–400	20–80	0.92	25– ≥ 400	25–200	0.99
FUM	40– ≥ 630	40–80	0.90	≤10– ≥ 600	25–200	0.99
GLN	40– ≥ 630	20–80	0.95	25– ≥ 1000	25–200	0.99
GLU	40– ≥ 630	40–80	0.73	25– ≥ 1000	25–100	0.98
ICI	≤ 10–400	10–80	0.99	25– ≥ 1000	25–150	0.98
LAC	≤ 10– ≥ 630	10–80	0.99	≤ 10– ≥ 400	25–100	0.99
LYS	60–500	NL	-	25–800	25–200	0.99
MET	60– ≥ 400	NL	-	25– ≥ 1000	25–200	0.99
MYR	40–400	40–80	0.91	25– ≥ 800	100–200	0.96
NAD+	nd	NL	-	25–1000	25–200	0.99
NADP+	nd	NL	-	25–1000	50–150	0.94
OAA	60–630	NL	-	50–2000	25–200	0.96
OIVAL	≤ 10– ≥ 630	10–80	0.99	≤ 10–1000	25–100	0.99
PAL	40–400	20–80	0.94	≤ 10– ≥ 1000	25–75	0.97
PEP	nd	NL	-	25– ≥ 1000	25–200	0.99
PHE	40– ≥ 630	40–80	0.71	≤ 10– ≥ 400	25–100	0.99
SUC	40- ≥ 630	40–80	0.91	≤ 10– ≥ 400	25–200	0.99
TRP	nd	NL	-	25–400	25–200	0.99
VAL	≤ 10– ≥ 630	10–80	0.98	≤ 10–200	25–200	0.99
2HB	≤ 10– ≥ 630	10–80	1.00	≤ 10– ≥ 1000	25–200	0.99

* Regression coefficient from calibration line within the linear range shown in the table. NL, non-linear; nd, not determined. See Table 1 for metabolite abbreviations.

On the other hand, the MCF derivatization seems not to be significantly affected by the sample matrix since the recovery of the MCF derivatives tended to be somewhat higher than for TMS derivatives (Figure 6). The internal standard L-alanine 2,3,3,3-d_4_ was recovered better when derivatized by MCF than by TMS (Figure 6), but its response factor was lower by 13% when spiked in a complex culture medium. This result shows that the efficiency of MCF derivatization is also affected by the components of the sample matrix. Nonetheless, a few metabolites were relatively better recovered by TMS than MCF derivatization (e.g.; 2-oxoglutarate; fumarate, lactate, 2-hydroxybutyrate) (Figure 6). They were mainly mono- and dicarboxylic acids. But all MCF derivatives presented recovery higher than 80% when spiked in a complex culture medium (Figure 6).

### Real biological samples

**Figure 7.**
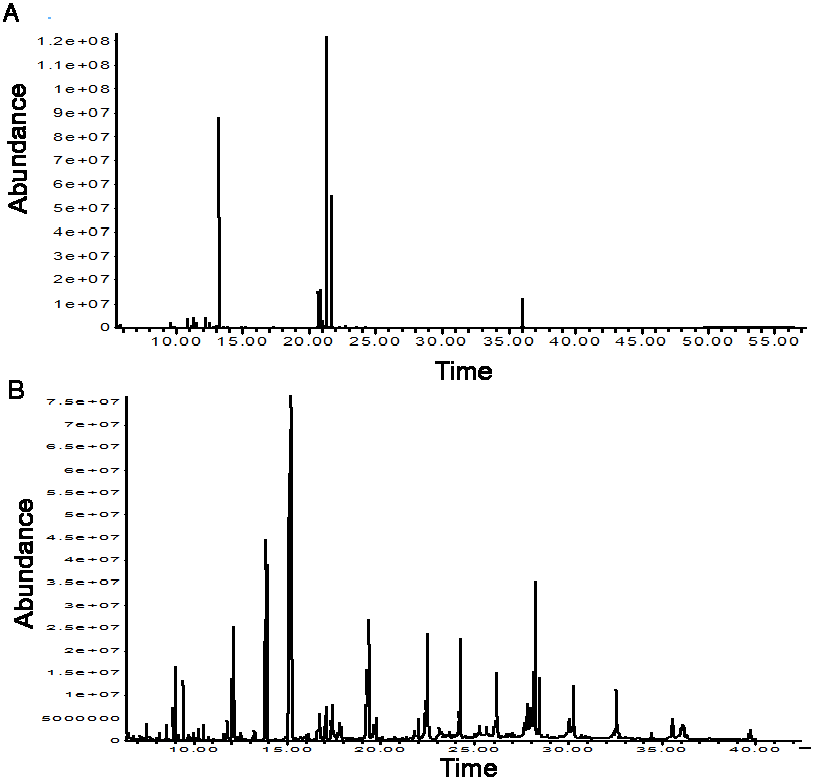
GC-MS chromatograms of derivatized extracellular metabolites from the same spent culture medium sample of *Acidovorax temperans*: A, sample derivatized by silylation reaction (TMS), and B, sample derivatized by alkylation reaction (MCF).

**Figure 8.**
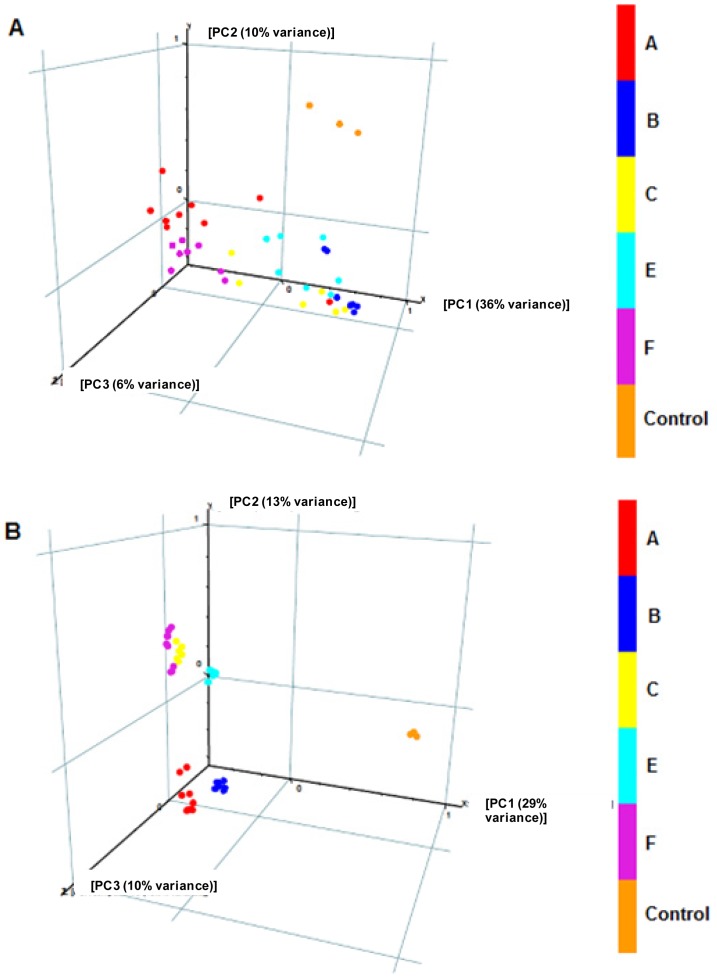
GC-MS extracellular metabolite data from samples of *Acidovorax temperans* (strains A-E). (**A**) samples derivatized by silylation reaction (TMS), and (**B**) samples derivatized by alkylation reaction (MCF). Principal component analysis (PCA) projection of extracellular metabolite data obtained from all detected compounds (deconvoluted raw chromatogram) from approximately 48 samples into a 3D space reveals distinct clustering of 5 data classes only in (B). Each sample (dots) was normalized by internal standard (d4-alanine) and only MS fragments related to metabolites with statistical significance (ANOVA) were considered. Control, uninoculated culture medium.

By using the same volume of samples we detected much less derivatized peaks after TMS derivatization than after MCF derivatization (Figure 7). Only 5 out of 26 amino and non-amino organic acids focused in this study were genuinely identified in TMS derivatized samples, while 15 were identified in MCF derivatized samples (Table 5). Consequently, poorer metabolite profiles obtained from TMS derivatization resulted in poorer discrimination power between different *A. temperans* strains (Figure 8A). MCF derivatization of spent culture of *A. temperans* clearly discriminated between different bacterial strains (except strain C and F, Figure 8B) and presented considerably better reproducibility (replicates clustering very closer to each other), suggesting it to be a more robust derivatization method for extracellular metabolomics than TMS.

**Table 5.** Some metabolites detected in the spent culture medium of *Acidovorax temperans* and their respective repeatability (RSD).

**Metabolite**	**RSD (n = 9)**	

	**Silylation (TMS)**	**Alkylation (MCF)**
ALA	18.9	7.1
ASP	ND	10.8
CYS	ND	5.9
FUM	79.5	7.0
ISO	33.1	0.01
LAC	ND	31.1
LEU	36.5	0.6
LYS	108	15.4
MET	ND	43.3
OAA	ND	18.2
OIVAL	ND	6.2
PAL	24.6	9.8
PHE	ND	8.6
SUC	ND	5.6
THR	8.2	4.9
TRP	ND	2.6
TYR	110.1	5.9
VAL	13.9	0.3

See Table 1 for metabolite abbreviations.

## Discussion

Although we observed very similar quality of metabolite identification based on the overall score of spectra using either derivatization technique, the analytical performance of TMS derivatization presented here is alarming, but consistent with other recent reports of unsatisfactory analysis of TMS derivatives of several amino acids (e.g. [9]). Contrary to the results reported by Koek *et al.* [7], we observed a very unsatisfactory analytical performance of several metabolites (amino and non-amino organic acids and nucleotides) derivatized by silylation. However, Koek *et al.* [7] applied a range of quality control measures, including a set of added deuterated standards to monitor extraction (phenylalaline-*d*_3_), lyophilization (glutamic acid-*d*_3_), derivatization (glucose-*d*_7_ and phenylalaline-*d*_5_) and GC-MS analysis (alanine- *d*_4_, dicyclohexylphthalate), and checking GC-MS performance by monitoring responses for standards. They reported that, in general, GC-inlet liners required changing after 20 samples had been injected, with occasional removal of a small section of the front end of analytical column to restore performance. With the strict quality control measures outlined, Koek *et al.* [7] claim the analytical performance of their TMS derivatization to be highly satisfactory with respect to stability, reproducibility, recoveries and linear ranges that meets requirements for target analysis in biological matrices. Data quality can be much improved where stable isotope standards are available (stable isotope dilution analysis: SIDA) [5], similar to our silylation performance for alanine (Figure 3 and Table 3). The results presented here represent the analytical performance of two derivatization methods corrected by one single internal standard (L-alanine 2,3,3,3-d_4_) and resolved in an uncut capillary column in use for over six months. The GC-inlet liner specific for each derivatization method was the same throughout the whole study. Whilst this may explain the poor results obtained for TMS derivatives, it reinforces the robustness of MCF derivatization.

It is important to emphasize, however, that we have targeted the most “unstable” classes of metabolites to be analyzed by silylation [1,5,18,19]. The formation of derivatives from MSTFA (TMS derivatizing reagent) involves the displacement of an N-methyltrifluoroacetamide leaving group by the analyte, and some metabolites provide equally good leaving groups. In this case, the derivatization reaction is only driven to product by the large excess of reagent, and the products are readily degradable. This is particularly the case for amides such as asparagine, and glutamine, and for thiols, and sulfonic derivatives, with the overall trend for ease of TMS derivatization and stability of products alcohol > phenol > carboxylic acid > amine > amide [1,2]. As shown by Koek *et al.* [7], the analysis of TMS derivatives of amines, and phosphoric functional groups shows intermediate variability of derivatization efficiencies (30–110%), and higher detection limits than for sugars and organic acids. Where a metabolite forms several TMS derivatives, reliability may be aided by maintaining a defined time window between derivatization and analysis and by summing the responses of all derivatives [9]; more complex procedures have been suggested to correctly weight the multiple peaks [8].

One additional important technical difference in the analysis of MCF derivatives was the wider dynamic and linear range of MCF derivatization compared to TMS. However, the samples derivatized by MCF were injected into the GC-MS under pulsed splitless injection while TMS derivatized samples were injected in split mode. Therefore, a considerably larger proportion of MCF sample reached the column compared to TMS samples, and this may explain the higher detection limit of some TMS derivatives. For TMS derivatized samples, splitless injection was not an attractive option in our experience because they usually contained a large amount of un-derivatized and non-volatile compounds as well as derivatizing reagents, which can rapidly damage the front end of the GC-capillary column. In addition, some metabolites are present at relative high concentrations in complex biological samples (e.g. sugars, urea, *etc.*) and their TMS derivatives are likely to overload the MS detector under splitless injection mode. Although sugars reduce the efficiency of MCF derivatization as shown in Figure 6 and also discussed in Villas-Bôas *et al.* [2], these compounds are not completely derivatized by alkylation reactions, and therefore, they are removed from the samples during the extraction of MCF derivatives with chloroform.

Silylation of organic compounds is the classical and most widely used derivatization procedure for metabolome analysis by GC-MS. Silylation is efficient for the analysis of alcohols (including sugars and derivatives), phenols and simple carboxylic acids such as mono and dicarboxylic acids and fatty acids. However, very stringent quality control measures such as the inclusion of several isotope labeled internal standards, replacement of inlet liner after every 20 injections and frequent removal (cutting) of the front end of the GC-column are required to achieve satisfactory results for many compounds [7]. Matrix-dependent variation in derivative volatilization on injection has been suggested by Noctor *et al.* [9] to be the likely source of problems. Evidently, MCF derivatives are less prone to such problems, and for the simultaneous analysis of polyfunctional amines, nucleotides and organic acids (mono-, di- and tricarboxylic acids; aromatic organic acids; keto and phospho-acids; and fatty acids) in complex biological samples such as microbial culture media (Figure 7), alkylation (MCF) derivatization reaction is more robust and hence more efficient in discriminating different microbial strains (Figure 8A). Ideally, MCF derivatization should be used in combination with TMS or any other silylation derivatization in order to gain a wider overview of cell metabolome.

## References

[1] Villas-Bôas S. G., Mas S., Åkesson M., Smedsgaard J, Nielsen J. (2005). Mass spectrometry in metabolome analysis. J. Mass Spectrom. Rev..

[2] Villas-Bôas S. G., Koulman A., Lane G. A., Nielsen J., Jewett M. C. (2007). Topics in Current Genetics—Metabolomics.

[3] Fiehn O., Kopka J., Dörmann P., Altmann T., Trethewey R. N., Willmitzer L. (2000). Metabolite profiling for plant functional genomics. Nat. Biotechnol..

[4] Roessner U., Wagner C., Kopka J., Trethewey R. N., Willmitzer L. (2000). Simultaneous analysis of metabolites in potato tuber by gas chromatography-mass spectrometry. Plant J..

[5] Gullberg J., Jonsson P., Nordstrom A., Sjostrom M., Moritz T. (2004). Design of experiments: an efficient strategy to identify factors influencing extraction and derivatization of *Arabidopsis thaliana* samples in metabolomic studies with gas chromatography/mass spectrometry. Anal. Biochem..

[6] Villas-Bôas S. G., Noel S., Lane G. A., Attwood G., Cookson A. (2006). Extracellular metabolomics: a metabolic footprinting approach to assess fiber degradation in complex media. Anal. Biochem..

[7] Koek M. M., Muilwijk B., van der Werf M. J., Hankemeier T. (2006). Microbial metabolomics with gas chromatography/mass spectrometry. Anal. Biochem..

[8] Kanani H. H., Klapa M. I. (2007). Data correlation strategy for metabolomics analysis using gas chromatography-mass spectrometry. Metabol. Eng..

[9] Noctor G., Bergot G., Mauve C., Thominet D., Lelarge-Trouverie C., Prioul J-L. (2007). A comparative study of amino acid measurement in leaf extracts by gas chromatography-time of flight-mass spectrometry and high performance liquid chromatography with fluorescence detection. Metabolomics.

[10] Kaspar H., Dettmer K., Gronwald W., Oefner P. J. (2008). Automated GC-MS analysis of free amino acids in biological fluids. J. Chromatogr. B..

[11] Qiu Y., Su M., Liu Y., Chen M., Gu J., Zhang J., Jia W. (2007). Application of ethyl chloroformate derivatization for gas chromatography-mass spectrometry based metabonomic profiling. Anal. Chim. Acta.

[12] Hušek P., Šimek P. (2006). Alkyl chloroformates in sample derivatization strategies for GC analysis. Review on a decade use of the reagents as esterifying agents. Curr. Pharmaceutical Anal..

[13] Villas-Bôas S. G., Delicado D. G., Åkesson M., Nielsen J. (2003). Simultaneous analysis of amino and non-amino organic acids as methyl chloroformate derivatives using gas chromatography-mass spectrometry. Anal. Biochem..

[14] Villas-Bôas S. G., Moxley J. F., Åkesson M., Stephanopoulos G., Nielsen J. (2005). High-throughput metabolic state analysis: the missing link in integrated functional genomics of yeasts. Biochem. J..

[15] Smart K. F., Aggio R. B. M., van Houtte J. R., Villas-Bôas S. G. (2010). Analytical platform for metabolome analysis of microbial cells using methyl chloroformate derivatization followed by gas chromatography–mass spectrometry. Nat. Prot..

[16] Förster J., Famili I., Palson B. Ø., Nielsen J. (2003). Genome-scale reconstruction of the *Saccharomyces cerevisiae* metabolic network. Genome Res..

[17] Attwood G. T., Reilly K., Patel B. K. C. (1996). *Clostridium proteoclasticum* sp. nov., a novel proteolytic bacterium from the bovine rumen. Int. J. Syst. Bacteriol..

[18] Broeckling C. D., Huhman D. V., Farag M. A., Smith J. T., May G. D., Mendes P., Dixon R. A., Sumner L. W. (2005). Metabolic profiling of *mendicago truncatula* cell cultures reveals the effects of biotic and abiotic elicitors on metabolism. J. Exp. Bot..

[19] Jiye A., Trygg J., Gullberg J., Johansson A. I., Johnsson P., Antti H., Marklund S. L., Moritz T. (2005). Extraction and GC-MS analysis of the human blood plasma metabolome. Anal. Chem..

